# Acute Changes in Striatal Microstructure Predict the Development of Interferon-Alpha Induced Fatigue

**DOI:** 10.1016/j.biopsych.2015.05.015

**Published:** 2016-02-15

**Authors:** Nicholas G. Dowell, Ella A. Cooper, Jeremy Tibble, Valerie Voon, Hugo D. Critchley, Mara Cercignani, Neil A. Harrison

**Affiliations:** * Brighton and Sussex Medical School, University of Sussex; † Department of Gastroenterology, Brighton & Sussex University Hospitals, Brighton; ‡ Department of Psychiatry, University of Cambridge; § Cambridge and Peterborough National Health Service Foundation Trust, Cambridge; ¶ Sackler Centre for Consciousness Science, University of Sussex, Falmer; ║ Sussex Partnership National Health Service Trust, Brighton, United Kingdom; ** Neuroimaging Laboratory, Santa Lucia Foundation, Rome, Italy

**Keywords:** Depression, Fatigue, Imaging, Inflammation, Insula, Interferon, Striatum

## Abstract

**Background:**

Interferon-alpha (IFN-α) is a key mediator of antiviral immune responses used clinically for hepatitis C treatment. Though effective, IFN-α induces marked behavioral changes that, when severe, can appear indistinguishable from major depression. Curiously, fatigue and motivational impairment evolve rapidly, suggesting acute engagement of immune-brain communicatory pathways, yet mood impairments typically emerge later, after weeks of treatment. Whether this reflects prolonged modulation of motivational processes underpinning fatigue or separate neurobiological mechanisms is currently unclear.

**Methods:**

Here, we used quantitative magnetization transfer (qMT) imaging, an advanced microstructural neuroimaging technique sensitive to effects of inflammation, in a prospective study design to measure acute brain changes to IFN-α and relate these to later development of discrete behavioral changes. Twenty-three patients initiating IFN-α treatment for hepatitis C underwent qMT imaging and blood sampling at baseline and 4 hours after their first IFN-α injection. Comprehensive behavioral and psychological assessments were completed at both scanning sessions and at treatment weeks 4, 8, 12, and 24.

**Results:**

IFN-α injection stimulated an acute inflammatory cytokine response and evoked fatigue that peaked between 4 and 12 weeks, preceding mood change by 4 weeks. In the brain, IFN-α induced an acute change in striatal microstructure that additionally predicted development of fatigue but not mood symptoms.

**Conclusions:**

Our findings highlight qMT as an in vivo biomarker of central effects of peripheral inflammation. We demonstrate exquisite sensitivity of the striatum to IFN-α, implicate striatal perturbation in IFN-α-induced fatigue, and dissociate this from mechanisms underlying IFN-α-induced mood symptoms, providing empirical support for distinct neural substrates mediating actions on motivation and mood.

Interferon-alpha (IFN-α) is a type I interferon released by specialized leucocytes (plasmacytoid dendritic cells) in response to viral stimulation (1) as well as virally infected cells and promotes a broader antiviral immune response. Externally administered IFN-α is also used clinically in the treatment of hepatitis C. Despite good clinical efficacy, direct and/or indirect actions on the brain result in often highly disabling behavioral changes including fatigue, mood, motivation, and cognitive impairments (2). When severe, these changes can appear indistinguishable from major depression and provide powerful empirical support for inflammatory theories of depression (3, 4). A striking feature of IFN-α-based treatment, though one rarely utilized experimentally, is that the impact on individual behavioral domains follows markedly different temporal trajectories. Changes in fatigue and motivation typically emerge within hours of the first IFN-α injection, suggesting the rapid engagement of immune-brain communicatory pathways and motivational processes. However, mood and cognitive effects are rarely prominent before 4 weeks of treatment, suggesting either a separate neurobiological mechanism or alternatively the secondary emergence of affective symptoms following prolonged modulation of motivational processes underpinning fatigue (2). Thus, the experimental investigation of early effects of IFN-α on the brain offers a unique window into the neurobiological mechanisms underlying IFN-α-induced depression, allowing the identification of neural processes that are acutely susceptible to IFN-α and predict the later emergence of discrete symptom clusters.

To date, most studies investigating the neurobiology of IFN-α-induced behavioral change utilize cross-sectional study designs, typically after 4 to 12 weeks of IFN-α treatment when the full spectrum of behavioral change is evident (5, 6, 7, 8). These provide important insights into the neural processes and structures susceptible to chronically administered IFN-α; however, their cross-sectional design limits the characterization of causal relationships between IFN-α-induced changes in the brain and the subsequent development of discrete behavioral changes that evolve with different temporal dynamics. In contrast, prospective studies enable the differentiation of changes induced by IFN-α from those resulting from the behavior itself. In one example, Capuron *et al.* (9) showed that acute reactivity of adrenocorticotropic hormone and cortisol to IFN-α injection can differentiate individuals who later develop depression. Further, by measuring the response well before the development of depression, they demonstrated this to be a key neurobiological process selectively engaged by IFN-α, rather than a consequence of the depression induced (which may alone cause hypothalamic-pituitary-adrenal axis hyperactivity) (10). Prospective studies investigating acute actions of IFN-α may also help identify and offer treatment to individuals most susceptible to the behaviorally impairing effects of IFN-α early in their treatment.

Here, we used a prospective study design to investigate the relationship between acute actions of IFN-α on the brain and subsequent behavioral change. We used quantitative magnetization transfer (qMT) imaging, an advanced structural magnetic resonance imaging (MRI) technique that exploits the phenomenon of magnetization transfer (MT) between free and macromolecular bound protons, to detect changes in microstructural environment. Molecules rich in hydroxyl and/or carboxyl groups appear to play a predominant role in MT (11). Though the precise molecules mediating MT change cannot be determined, it is noteworthy that metabolites such as lactate (which contains a hydroxyl and carboxyl group) as well as pH have previously been implicated (12, 13). qMT has also been shown previously to be sensitive to the central effects of peripheral inflammation in both rodents (14, 15) and humans (16).

We recruited 23 patients initiating IFN-α-based treatment for hepatitis C infection and followed them over their 6-month duration of treatment. Of these patients, 19 completed repeat qMT imaging at both baseline and 4 hours after their first IFN-α injection. Blood samples were obtained immediately after both scanning sessions to characterize the profile of cytokine changes induced acutely by IFN-α. Comprehensive clinical assessments were completed at both scanning sessions and at 4, 8, 12, and 24 weeks of treatment to quantify and characterize symptoms of fatigue and depression.

Key aims were to determine first whether IFN-α induces acute microstructural reorganization within the brain and second whether the pattern of evoked changes provides evidence for activation of an indirect (neurally mediated) or direct immune-brain communicatory pathway. We next aimed to investigate whether acute changes in brain microstructure also predict the later development of fatigue and motivational change. Finally, we tested if acute changes within systems supporting motivational behavior (and linked to expression of fatigue) additionally contributed to the later development of mood change. A subaim was to further characterize the nature of IFN-α-induced fatigue, in particular its relationship to subjective sleepiness or the propensity to fall asleep.

Unlike the reported central response to inflammation induced using bacterial antigens (16, 17, 18, 19, 20), the human literature concerning response to chronic IFN-α provides little support for engagement of typical interoceptive pathways to insula (5, 6, 21). Instead, there appears to be a particular sensitivity of striatal structures. It is currently unclear whether this reflects habituation of interoceptive pathways during chronic IFN-α treatment or, alternatively, more direct actions of IFN-α on subcortical structures as suggested by rodent studies (22, 23). To address this, we investigated the acute effects of IFN-α on bilateral insula (the cortical terminus of human interoceptive pathways) (24, 25) and the striatum, a structure sensitive to chronic IFN-α but not typically implicated in neurally mediated interoception (24). Given the acute onset of fatigue and motivational impairment, we predicted that acute changes in ventral striatal microstructure would additionally predict the evolution of fatigue but not necessarily later mood symptoms.

## Methods and Materials

### Participants

Twenty-three individuals (17 male subjects, mean 48.8 ± 10.9 years) initiating IFN-α-based therapy for hepatitis C were recruited. All were fluent in English, aged 18 to 64 years, and fulfilled National Institute for Health and Care Excellence guidelines for starting IFN-α-based therapy. Participants had a baseline psychiatric evaluation of current mental state and previous psychiatric history, using the Mini-International Neuropsychiatric Interview (M.I.N.I.) (26). Participants were excluded if they were receiving treatment for depression at study enrollment, had a history of psychotic or autoimmune illness, had not abstained from substance abuse for at least 6 months, were co-infected with human immunodeficiency virus, or had any cause for liver disease other than hepatitis C. The study was approved by the Cambridge Central National Research Ethics Committee. All subjects provided written informed consent.

### Study Design

The study utilized a prospective cohort design. Participants were evaluated at baseline (mean 7 days before treatment), 4 hours after their first IFN-α injection, and weeks 4, 8, 12, and 24 of IFN-α-based therapy. Psychopathological symptoms were evaluated at each visit using the Profile of Mood States (POMS) questionnaire (27), Epworth Sleepiness Scale (ESS) (28), fatigue visual analog scale (fVAS), Hamilton Depression Rating Scale (HAMD), State and Trait Anxiety Inventory (STAI), and M.I.N.I. MRI followed by blood sampling, blood pressure, and temperature was repeated at baseline and 4 hours after the first IFN-α injection to index acute effects of IFN-α on brain microstructural environment and circulating cytokines, respectively. Of the total cohort, 19 participants (14 male participants, mean 44.4 ± 10.7 years) completed both MRI sessions and 20 participants (17 male participants, mean 49.6 ± 11.2 years) completed both blood samples. One female participant was later excluded from the image analysis due to metal-induced artifact. All participants completed all clinical evaluations.

### Behavioral Analyses

Effects of IFN-α on global fatigue were measured using the fVAS and fatigue subcomponents of tiredness, vigor, and subjective sleepiness with the POMS and ESS. Actions on depressive and anxiety symptoms were additionally recorded using the M.I.N.I., HAMD, and STAI. Effects of IFN-α on all psychopathological symptoms and the relationship between different behavioral domains were analyzed in SPSS 21.0 (IBM Corp., Armonk, New York) using repeated-measures analyses of variance and subsequent paired sample *t* tests or regression analyses, respectively. Mauchly’s sphericity test was performed, and results reported followed Greenhouse-Geisser correction of degrees of freedom where appropriate.

### Cytokine Analyses

Blood (20 mL) was drawn into Vacutainer tubes (Becton and Dickinson, Franklin Lakes, New Jersey) containing ethylenediaminetetraacetic acid anticoagulant then centrifuged at 1250*g* for 10 minutes. Plasma was removed, aliquoted, and frozen at −80°C before analysis. Plasma IFN-α was measured using high-sensitivity VeriKine ELISA (Human IFN Alpha Multi-Subtype ELISA Kit (TCM); PBL Assay Science, Piscataway, New Jersey). Interleukin-6 minimum detectable dose (MDD) = .039 pg/mL, tumor necrosis factor (TNF) MDD = .106 pg/mL, interleukin-1β MDD = .057 pg/mL, and interleukin-10 MDD = .09 pg/mL for the high-sensitivity Quantikine ELISAs (R&D Systems, Abingdon, United Kingdom) and interleukin-1 receptor antagonist MDD = 6.3 pg/mL for the Quantikine ELISA.

### MRI

MRI was performed on a 1.5T Siemens Avanto (Erlangen, Germany), equipped with a 32-channel phased-array receive-only head coil. qMT data were acquired using the balanced steady-state free precession method (29) with a three-dimensional true fast imaging with steady-state precession sequence (matrix = 256 × 96, slices = 32, slice thickness = 5 mm). A total of 22 volumes were acquired (flip angle varied between 5° and 40°, pulse duration between .2 and 2.5 ms) resulting in a range of repetition time (TR) from 3.66 ms to 5.96 ms and echo time (TE) from 1.83 ms to 2.98 ms. T1 mapping was performed by acquiring three gradient echo volumes (flip angles = 5°, 15°, and 25°; TR = 30 ms; TE = 5 ms) with matched matrix size and field of view. A high-resolution T1-weighted anatomical scan was acquired using a magnetization prepared rapid acquisition gradient-echo (TR = 1160 ms, TE = 4.24 ms, inversion time = 600 ms, matrix = 256 × 256, slices = 192, slice thickness = 1 mm, flip angle = 15°). Other acquisitions including functional MRI were additionally acquired and will be reported separately.

Participant-specific qMT and T1 mapping volumes were spatially realigned to their respective anatomical volume using SPM8 rigid-body registration (Wellcome Trust Centre for Neuroimaging, University College London, United Kingdom; http://www.fil.ion.ucl.ac.uk/spm/). The qMT parameters forward exchange rate constant (k_f_), T_2_ of free water component (T_2f_), and bound proton fraction (F) were then extracted in a voxelwise manner using Levenberg-Marquardt nonlinear least squares fitting to the binary spin bath model (29). T1 maps were obtained through voxelwise fitting of the data to theoretical pixel values for the spoiled gradient echo for the three flip angles. Symmetric diffeomorphic mapping using Advanced Normalization Tools (http://stnava.github.io/ANTs) was applied to the magnetization prepared rapid acquisition gradient-echo images to generate a group-specific brain template (30). qMT parameter maps were deformed to this template, transformed into Montreal Neurological Institute space, and then smoothed with an 8-mm^3^ full width and half maximum Gaussian kernel. Voxelwise paired *t* tests were used to identify acute effects of IFN-α on regional k_f_, T_2f_, and F parameters.

Finally, we performed regression analyses to investigate whether acute actions of IFN-α on regional brain microstructural environment additionally predicted the subsequent development of fatigue or mood change. Baseline parameter maps were subtracted from their respective maps at 4 hours and then regressed against changes in fatigue and mood. To minimize variance induced by changes in medical management, e.g., starting an antidepressant, we restricted this analysis to changes at 4 weeks only. No participant had any change in prescribed medication at this time. We restricted these correlations with k_f_ and T_2f_ to a priori regions of interest (ROIs) in the ventral striatum and insula.

### ROIs

We defined four a priori ROIs for analyzing the main effects of IFN-α: left and right striatum and insula. Results are additionally reported for striatal subcomponents: putamen and caudate. Masks for each ROI were produced using the WFU Pickatlas (http://fmri.wfubmc.edu/software/PickAtlas) (31). Values of k_f_ and particularly T_2f_ in cerebrospinal fluid (CSF) (~300 ms) are very different from those in tissue (~40 to 70 ms). As a result, these parameters are extremely sensitive to CSF contamination near the ventricles. To avoid biasing the mean of k_f_ and T_2f_ values, we excluded voxels with implausibly high T_2f_ values (>150 ms) using a subject specific masking procedure. Furthermore, since this was an ROI analysis, it was not necessary to use smoothed T_2f_ and k_f_ parameter maps, avoiding introducing further partial volume effects. Mean changes in k_f_ and T_2f_ within all mask voxels were extracted from the insula and ventral striatum (32) for each participant using FSL (FMRIB, Oxford, United Kingdom; http://fsl.fmrib.ox.ac.uk) and then used to investigate the relationship with subsequent changes in fatigue and mood.

### Multiple Comparisons

Whole-brain corrected cluster significance was determined using stringent familywise error (FWE) correction. Only clusters surviving a FWE cluster-corrected threshold of α < .05 are reported for whole-brain analyses.

## Results

### Inflammatory Cytokine Response to IFN-α

**Figure 1:**
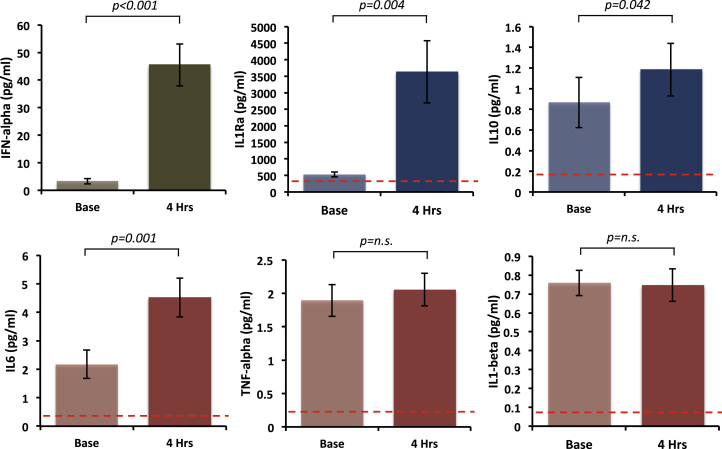
Cytokine response to interferon (IFN)-alpha injection. Mean plasma cytokine concentrations before (Base) and 4 hours after the first subcutaneous injection of pegylated interferon-α-2a. Error bars represent standard error of the mean. IL1, interleukin-1; IL1Ra, interleukin-1 receptor antagonist; IL6, interleukin-6; IL10, interleukin-10; n.s., nonsignificant; TNF, tumor necrosis factor.

Initial IFN-α injection was associated with ~fourteenfold increase in plasma IFN-α (from mean ± SE) (3.12 ± .95 pg/mL at baseline to 43.26 ± 7.77 pg/mL at 4 hours, *t*_19_ = 5.12, *p* < .001) (Figure 1). We also observed a twofold increase in interleukin-6 (2.13 ± 2.18 pg/mL to 4.31 ± 3.11 pg/mL, *t*_19_ = 3.86, *p* = .001). Plasma TNF and interleukin-1β were not significantly altered at this time point (1.89 ± .24 pg/mL to 2.05 ± .24 pg/mL, *t*_19_ = 1.45, *p* = .164, and .76 ± .065 pg/mL to .74 ± .084 pg/mL, *t*_19_ = .27, *p* = .79), though there was a marked increase in interleukin-1 receptor antagonist from 526.76 ± 74.32 pg/mL to 3630.75 ± 938.82 pg/mL (*t*_19_ = 3.32, *p* = .004) and more moderate increase in interleukin 10 from .83 ± .24 pg/mL to 1.13 ± .25 pg/mL (*t*_19_ = 2.18, *p* = .042) demonstrating a broader proinflammatory and anti-inflammatory cytokine response to IFN-α (Figure 1).

Exploratory multiple-regression analysis between acute changes in plasma cytokines and fatigue (4 weeks minus baseline) identified no significant association between any mediator of the peripheral inflammatory response and subsequent development of fatigue (*p* > .05).

### Psychological Effects of IFN-α-Based Treatment

**Figure 2:**
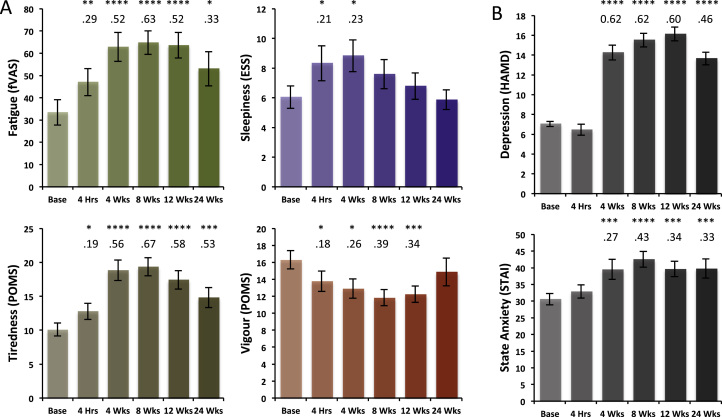
Interferon-alpha (IFN-α)-induced changes in fatigue and mood. **(A)** Change in global fatigue (fatigue visual analog scale [fVAS]) and fatigue subcomponents of tiredness and vigor (Profile of Mood States [POMS] subscales) and sleepiness (Epworth Sleepiness Scale [ESS]) during the 24 weeks of treatment with INF-α. **(B)** Change in depression (Hamilton Depression Rating Scale [HAMD]) and state anxiety (State and Trait Anxiety Inventory [STAI]) during the 24 weeks of treatment with INF-α. Base denotes baseline scores. Error bars show the standard error. Stars denote associated *p* values: *p < .05, **p < .01, ***p < .005, ****p < .001. Numbers below denote associated effect sizes (η^2^).

IFN-α treatment showed a strong effect on global fatigue (fVAS, *F*_5,110_ = 10.01, *p* < .001) increasing from 33.43 ± 5.57 to peak 64.74 ± 5.01, effect size (η^2^) = .63 at 8 weeks (Figure 2A). This increase in fatigue was rapid, with a moderate effect (η^2^ = .29) already observed at 4 hours (*t*_19_ = 2.96, *p* = .007) demonstrating acute sensitivity to peripheral IFN-α (Figure 2A). Analysis of fatigue subcomponent scores demonstrated a similar profile of changes and effect sizes for the POMS tiredness and vigor subscales. Modest effects were also observed on sleep propensity (ESS, *F*_5,110_ = 3.19, *p* = .023) (Figure 2A). Together, these findings suggest a large effect of IFN-α on global fatigue predominantly mediated through an increase in feelings of tiredness and to a lesser extent reduced vigor. Though associated with a modest increase in sleep propensity, this contribution was relatively weak and short-lived and did not persist throughout treatment. As previously reported, IFN-α-based therapy had a large effect on HAMD depression symptoms (*F*_5,110_ = 20.27, *p* < .001) with significant effects observed from 4 weeks until the end of treatment (Figure 2B). Moderate effects following a similar trajectory were also observed for state (*F*_5,110_ = 6.64, *p* < .001) though not trait STAI anxiety (*F*_5,110_ = 2.46, *p* = .066).

Acute changes in global fatigue (fVAS) weakly predicted the increase in fatigue experienced at 4 weeks (*R*^2^ = .125, *p* = .052). However, no comparable association between acute and subsequent mood change was observed (*R*^2^ = .00, *p* > .1) or any association between acute change in fatigue and subsequent mood change (*R*^2^ = −.05, *p* > .1).

### Acute Effects of IFN-α on qMT Imaging

**Figure 3:**
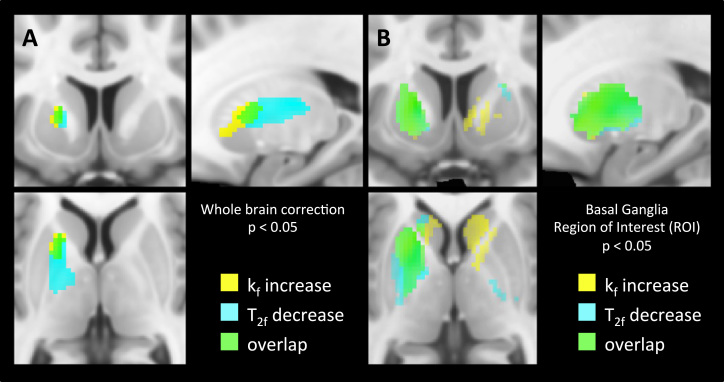
Interferon-alpha-induced changes in quantitative magnetization transfer parameters. Changes in quantitative magnetization transfer parameters k_f_ and T_2f_ 4 hours after commencing interferon-alpha-based treatment compared with baseline. Voxels showing a significant increase in forward exchange rate constant (k_f_) are shown in yellow and voxels showing a significant decrease in T_2_ of free water component (T_2f_) are shown in cyan. **(A)** Whole brain analysis showing the left striatal clusters surviving stringent familywise error correction at *p* < .05. **(B)** A priori region of interest analysis showing changes in k_f_ and T_2f_ for all voxels within the striatal region of interest at *p* < .05.

Initial whole-brain analysis using stringent FWE cluster-correction identified a single left striatum centered cluster associated with an IFN-α-induced increase in k_f_ (cluster size = 293, FWE *p* = .043, peak Z = 4.02; Figure 3A) and a single similarly located left striatal cluster showing a complementary decrease in T_2f_ (cluster size = 282, FWE *p* = .030, peak Z = 4.54; Figure 3A). No other brain region demonstrated a significant change in either k_f_ or T_2f_ following IFN-α at this stringent threshold. For T_2f_, this cluster was tightly constrained to the basal ganglia, though for k_f_ it extended to include the left anterior insula (−28, 24, −8) (Figure 3A).

To investigate this further, we next examined the effect of IFN-α on mean parameter values within each of our preplanned striatum and insula ROIs. Of note, these ROIs were carefully constructed to avoid potential biasing of findings by CSF partial volume effects. This analysis confirmed the complementary changes in k_f_ and T_2f_ in the left striatum observed in our whole-brain analysis (mean k_f_ increase: .071 s^−1^, *t*_1,17_ = 3.50, *p* = .003, and mean T_2f_ decrease: 1.29 ms, *t*_1,17_ = 3.04, *p* = .007, respectively) with changes evident in both putamen (k_f_: *p* = .012, T_2f_: *p* = .012) and caudate subregions (k_f_: *p* = .032, T_2f_: *p* = .021). However, it additionally identified similar though statistically weaker changes in mean k_f_ and T_2f_ in the left insula (mean k_f_ increase: .053 s^−1^, *t*_1,17_ = 3.45, *p* = .003, mean T_2f_ decrease: .76 ms, *t*_1,17_ = 2.16, *p* = .045) and a significant increase in k_f_ within the right striatum (mean change: .057 s^−1^, *t*_1,17_ = 2.42, *p* = .027) (Figure 3B; Table 1). The subsidiary parameter F (bound proton fraction) was unchanged.

**Figure 4:**
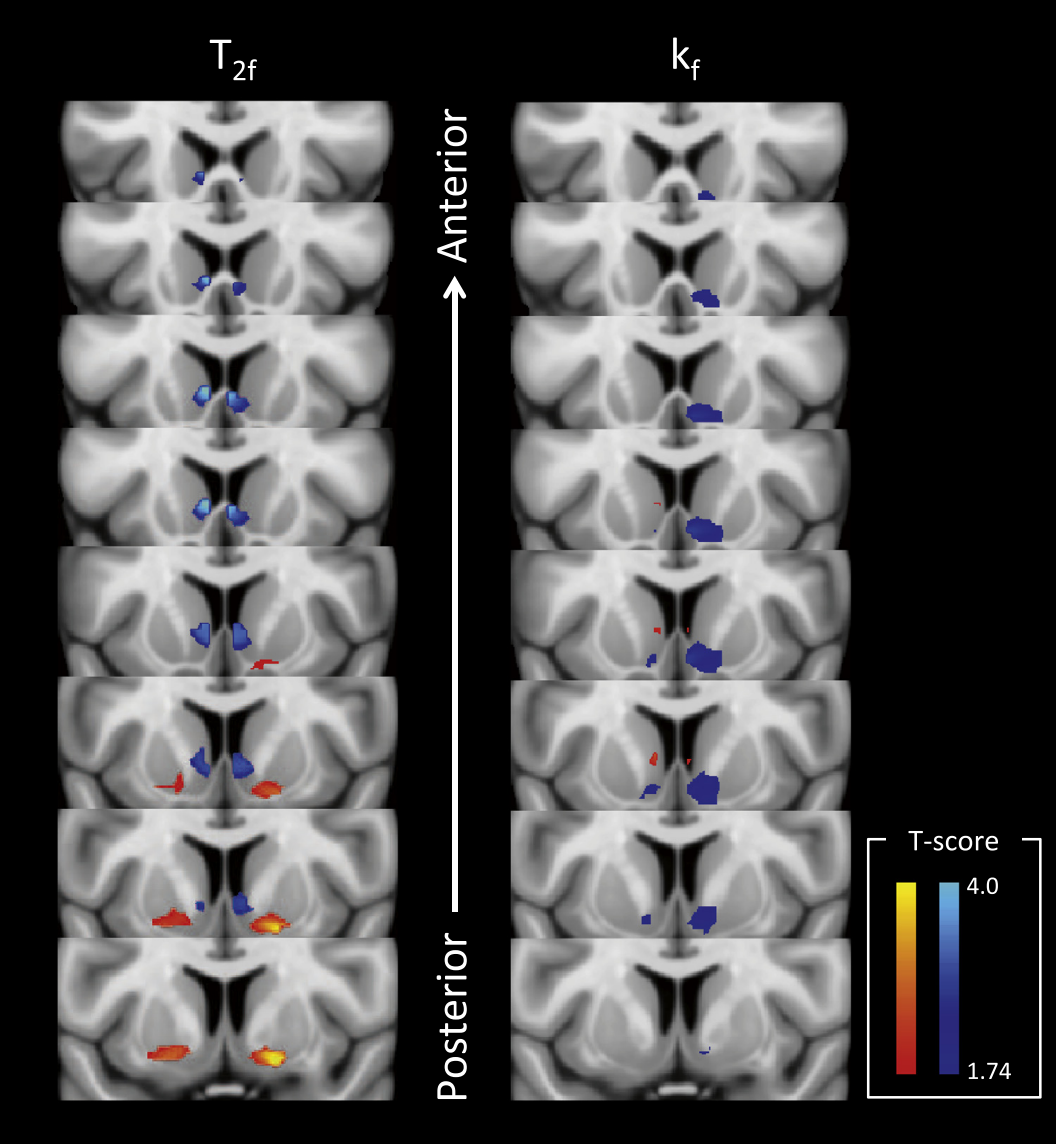
Left side shows voxels where acute changes in T_2f_ significantly predict the increase in fatigue at 4 weeks and the right side voxels where acute changes in k_f_ significantly predict the increase in fatigue at 4 weeks. Slices illustrate changes across the whole of the ventral striatum region of interest (from posterior to anterior). Blue to light blue denotes negative correlation and red to yellow positive correlation with associated *t*-scores denoting equivalent *p* values (*p* = .05 to *p* = .0004).

To investigate whether these acute changes in striatal and insula magnetization exchange additionally predicted the later development of fatigue, we next performed a correlational analysis of acute changes in k_f_ and T_2f_ and changes in fatigue experienced 4 weeks later. This analysis focused specifically on ventral striatal and insula ROIs where IFN-α and typhoid vaccine-induced changes in glucose metabolism, respectively, have been shown to correlate with simultaneous change in fatigue (5, 19). This analysis demonstrated striking correlations bilaterally between shifts in k_f_ and T_2f_ within the ventral striatum and the subsequent development of fatigue (Figure 4). However, no similar association was observed for the insula and similar analyses exploring the associations between these ROIs and change in mood (HAMD) were negative.

## Discussion

Here, we used qMT, an advanced microstructural MRI technique, to show that IFN-α induces a rapid and selective change in striatal molecular structure, a region previously shown to be metabolically and neurochemically sensitive to chronic IFN-α administration and strongly implicated in concomitant motivational change (5, 21). Specifically, we observed an increase in the rate of MT from free (water) to molecular-bound protons (k_f_) and a complementary reduction in free water spin-spin relaxation (T_2f_) within the striatum 4 hours after IFN-α injection. The functional significance of this acute change in microstructural environment was further supported by our between-subject analysis, which demonstrated that acute actions of IFN-α on ventral striatal MT were sufficient to predict fatigue experienced 4 weeks later. Together, they demonstrate an exquisite sensitivity of basal ganglia structures to acute changes in peripheral IFN-α, which play a potentially etiological role in the later development of fatigue. Interestingly, we also observed more constrained changes within the insula, suggesting that the cortical substrate for the representation of many aspects of bodily physiology (24, 33) is also sensitive to acute changes in IFN-α. Importantly, however, unlike the response observed in the striatum, these changes did not predict subsequent development of fatigue, suggesting that insula has a less prominent role in mediating cognitive and behavioral symptoms evoked by IFN-α. Additionally, the lack of a predictive association between acute changes in the striatum (or insula) and later depressive symptoms provides empirical support for the presence of distinct neural substrates mediating actions of IFN on motivation and mood/cognition.

Our findings also extend the earlier evidence for strikingly localized changes in the neurochemistry of the striatum after chronic IFN-α. This evidence includes bilateral (though left predominant) increases in striatal ^18^fluoro-deoxy-glucose uptake (with left ventral striatum changes additionally correlating with fatigue) (5); left-sided, but not right-sided, increases in striatal glutamate/creatine ratio (which correlated with IFN-α-induced motivational impairment) (21); and increased striatal ^18^fluorodopa uptake (with changes in caudate additionally correlating with fatigue) (6). We used qMT to measure the exchange of magnetization between free (water) and bound proton pools and provide an indirect quantification of hydrophilic molecules rich in hydroxyl, amine, and carboxyl groups (11). Though qMT cannot identify the precise molecules underlying this change, previous data linking changes in k_f_ to alterations in lactate (which contains a hydroxyl and carboxyl group) and local pH (12, 13) suggest a likely metabolic change driving these effects. Taken together, these convergent data from four different neuroimaging techniques strongly implicate the actions of IFN-α on the striatum in the etiology of IFN-induced fatigue.

Interestingly, studies where inflammation is induced using bacterial antigens (16, 19, 20) or inhaled antigens that induce an allergic type response (21) often report prominent changes in a human interoceptive pathway projecting to insula, which also may correlate with evoked fatigue. However, in our current study, we identified relatively modest changes within this pathway, which is implicated in providing a cortical representation of bodily state across physiologic domains including inflammation (24, 33). This suggests that visceral afferents may not be the principle pathway mediating the central effects of IFN-α to engender experience of fatigue. Alternative potential mechanisms include direct actions of IFN-α on the brain or actions of downstream mediators such as cytokines or prostaglandins produced either peripherally or at the endothelium. Though we cannot conclusively address this issue with our current study, the remarkably acute nature of these neurobehavioral changes (occurring 4 hours after IFN-α), as well as relatively modest changes in other circulating cytokines observed at this time, point toward a direct action of IFN-α.

Supporting this interpretation, CSF concentrations of IFN-α have been shown to be markedly elevated in humans after 12 weeks of IFN-α therapy and a threefold increase in CSF IFN-α observed in rhesus monkeys from 3 hours after IFN-α injection (34). Like peripheral mononuclear cells (35), mouse basal ganglia and hippocampal neurons show marked sensitivity to locally administered IFN and upregulate hundreds of IFN-stimulated genes within hours of administration (22). Further, rodent studies have also reported profound central nervous system induction of IFN-inducible genes within hours of intraperitoneal injection of even modest amounts of mouse IFN-α (two orders of magnitude lower than typical human treatment doses) (23), indicating that central nervous system cell populations are highly sensitive to IFN-α within the acute time frames investigated here. Nevertheless, to date, no saturable transport system for IFN-α has been described (36). Thus, the appearance of IFN-α within the CSF following peripheral injection suggests that IFN-α either enters the brain via passage through leaky regions in the blood-brain barrier or alternatively activates cells spanning the blood-brain barrier to induce central IFN-α production.

Why the striatum should be so sensitive to peripherally administered IFN-α remains unclear. Though it is interesting to note that fatigue is a prominent symptom of other diseases that affect the basal ganglia (37). The basal ganglia are also exquisitely vulnerable to multiple neurodegenerative processes and hypoxic injury, as well as direct viral invasion (37). Neurons, including those in the basal ganglia, with a high turnover of neurotransmitter proteins may also be more sensitive to processes such as ISGylation, which downregulates the function of host proteins following IFN-α exposure, particularly those that are newly synthesized (38).

In our current study, we also show that acute changes in ventral striatal microstructure differentiate individuals most susceptible to the motivationally impairing effects of chronic IFN-α. Specifically, we identified a spatial gradient to this association with bilateral posterior regions showing a strong positive association with motivational change and bilateral anterior regions showing a negative association. Interestingly, this finding accords well with human and rodent data that implicate the ventral striatum in the learned control of behavior in the face of rewards and punishments. For example, Seymour *et al.* (39) localized appetitive prediction error to more anterior regions than aversive prediction errors. This anterior-posterior gradient also resembles that seen in stimulation studies of the ventral striatum in rats, in which microinjection of a gamma-aminobutyric acid agonist (or glutamate antagonist) into more anterior regions produces appetitive responses (feeding) and into more posterior regions produces aversive responses (paw treading, burying) (40, 41, 42). These studies are characteristic of a growing body of evidence pointing to a role of the ventral striatum in motivation with distinct neuronal responses associated with appetitive and aversive events (43, 44, 45, 46, 47).

As reported for inflammation induced with naturalistic inflammatory challenges (19), acute changes in circulating levels of IFN-α or other measured proinflammatory or anti-inflammatory cytokines did not show any predictive value for the later development of fatigue. This provides further support for the proposition that interindividual differences in sensitivity to the central effects of inflammation, rather than their peripheral levels, are likely to be most critical in determining subsequent behavioral change (48). Indeed, the only association that could be identified was between baseline fatigue scores and their subsequent change, which is consistent with effects of response bias (the tendency to over/underreport symptoms).

A caveat for our current study is that we focused on acute responses to IFN-α; it is currently unclear whether more prolonged exposure to IFN-α results in similar MT changes in brain regions beyond the striatum, including potentially, brain structures involved in the development of depression symptoms. Similarly, whether the MT changes persist though treatment or relate to chronic fatigue occasionally experienced even after completion of IFN-α-based therapy remains to be resolved. The relatively modest sample size is a potential limitation of our current study. However, our use of an efficient within-subject design together with findings of altered striatal MT parameters, even when averaged across all ROI voxels, as well as at a stringent whole-brain FWE cluster correction level, support the robustness of the results reported.

To conclude, we show that IFN-α rapidly alters striatal microstructural environment, an action that is sufficient to predict the development of fatigue 4 weeks later. This highlights the acute sensitivity of striatal structures to peripherally administered IFN-α and strongly implicates them in the etiology of IFN-α-induced fatigue and motivational change. A lack of association with mood change further supports the position that actions on discrete behavioral components result from actions on different neural substrates.

## Figures and Tables

**Table 1:** Changes in k_f_ and T_2f_ Within Basal Ganglia ROIs Measured 4 Hours After IFN-α

Side	Region	Change in k_f_ (s^−1^)	*p* Value	Change in T_2f_ (ms)	*p* Value
Left	Striatum	.071	.003^a^	−1.29	.009^a^
	Putamen^b^	.043	.012^a^	−1.20	.012^a^
	Caudate^b^	.170	.032^a^	−1.73	.021^a^
	Insula	.053	.003^a^	−.76	.045^a^
Right	Striatum	.057	.027^a^	−.74	.266
	Putamen^b^	.025	.250	−.83	.187
	Caudate^b^	.167	.024^a^	−.46	.642
	Insula	.011	.592	−.40	.315

IFN-α, interferon-alpha; k_f_, forward exchange rate constant; ROI, region of interest; T_2f_, T_2_ of free water component.

a Statistically significant results.

b Subregions that make up the striatum.
